# The coherence of macrocirculation, microcirculation, and tissue metabolic response during nontraumatic hemorrhagic shock in swine

**DOI:** 10.14814/phy2.13216

**Published:** 2017-04-11

**Authors:** Halvor Langeland, Oddveig Lyng, Petter Aadahl, Nils‐Kristian Skjærvold

**Affiliations:** ^1^ Department of Anesthesiology and Intensive Care MedicineTrondheim University HospitalTrondheimNorway; ^2^ Department of Circulation and Medical ImagingFaculty of MedicineNorwegian University of Science and Technology (NTNU)TrondheimNorway; ^3^ Unit of Comparative MedicineFaculty of MedicineNorwegian University of Science and Technology (NTNU)TrondheimNorway

**Keywords:** Hemorrhagic shock, microcirculation, microdialysis, microspheres, pig, swine

## Abstract

Hemorrhagic shock is clinically observed as changes in macrocirculatory indices, while its main pathological constituent is cellular asphyxia due to microcirculatory alterations. The coherence between macro‐ and microcirculatory changes in different shock states has been questioned. This also applies to the hemorrhagic shock. Most studies, as well as clinical situations, of hemorrhagic shock include a “second hit” by tissue trauma. It is therefore unclear to what extent the hemorrhage itself contributes to this lack of circulatory coherence. Nine pigs in general anesthesia were exposed to a controlled withdrawal of 50% of their blood volume over 30 min, and then retransfusion over 20 min after 70 min of hypovolemia. We collected macrocirculatory variables, microcirculatory blood flow measurement by the fluorescent microspheres technique, as well as global microcirculatory patency by calculation of Pv‐aCO_2_, and tissue metabolism measurement by the use of microdialysis. The hemorrhage led to anticipated changes in macrocirculatory variables with a coherent change in microcirculatory and metabolic variables. In the late hemorrhagic phase, the animals' variables generally improved, probably through recruitment of venous blood reservoirs. After retransfusion, all variables were normalized and remained same throughout the study period. We find in our nontraumatic model consistent coherence between changes in macrocirculatory indices, microcirculatory blood flow, and tissue metabolic response during hemorrhagic shock and retransfusion. This indicates that severe, but brief, hemorrhage with minimal tissue injury is in itself not sufficient to cause lack of coherence between macro‐ and microcirculation.

## Introduction

Hemorrhagic shock is a pathological condition where intravascular blood volume, and thus tissue oxygen delivery is reduced to a level where the tissues' metabolic requirements are compromised. Acute hemorrhage is compensated in the organism by an increase in heart rate in an effort to maintain cardiac output, enhanced oxygen extraction, and later a redistribution of the remaining blood volume from the nonvital organs (splanchnic, renal, muscles, and skin) to the vital organs (brain and heart) (Vincent and De Backer 2014).

While hemorrhagic shock is caused by an alteration in the macrocirculation, and clinically monitored as changes in macrocirculatory indices, the pathophysiological important consequence of the shock state is on the microcirculatory level with cellular asphyxia caused by diminished oxygen delivery to the tissues.

Experimental and clinical studies of shock states, where the inflammatory response is prominent (e.g., septic shock), find lack of coherence between macro‐ and microcirculatory changes, and it is especially emphasized that the microcirculatory changes take place before the macrocirculatory deterioration, and sustains even after macrocirculatory restoration (Spronk et al. 2004; De Backer et al. 2014; Vincent and De Backer 2014). Thus, resuscitation based on macrocirculatory variables alone does not give reassurance of reestablished adequate microcirculation (Ince 2004). Weather this incoherence also is present in “pure” hemorrhagic shock is still uncertain as the hemorrhagic shock often includes a “second hit” by tissue trauma (Van Iterson et al. 2012; Tachon et al. 2014), which can induce an inflammatory response on its own (Lord et al. 2014).

In order to study the (patho‐) physiologic integration between macrocirculatory, microcirculatory, and tissue metabolic changes, all three different spatial levels should be examined simultaneously. Due to the lack of biosensing opportunities capable of monitoring changes on a cellular level within humans, the use of animal models is mandated. Therefore, we combined several different biosensing techniques in a swine model to detect hemodynamic changes during a controlled hemorrhagic shock with minimal trauma; classical macrocirculatory monitoring (invasive systemic and pulmonary blood pressure, arterial and mixed venous blood gas, and ECG), regional blood flow (fluorescent‐labeled polystyrene microspheres), as well as global microcirculatory patency by calculation of the venous‐to‐arterial carbon dioxide difference (Pv‐aCO_2_) (Ospina‐Tascón et al. 2016a), and regional metabolism (microdialysis). We focused on the nonvital organs (kidney, gut, and muscle), as we predicted would be the areas with most pronounced change.

It follows from the argumentation above that it is still uncertain to what extent the hemorrhage itself contributes to this lack of circulatory coherence, and we hypothesize that a “pure” hemorrhagic shock with minimal tissue trauma would keep coherence between metabolism, macro‐, and microcirculatory indices.

## Materials and Methods

### Ethical approval

The Norwegian State Commission for Animal Experimentation approved this study (FOTS‐number 6539).

### Animal handling

Nine immature outbred swine (*Sus scrofa domesticus*; Norwegian Landrace 50%, Yorkshire 25%, and Duroc 25%) of male gender (range: 27–36 kg) were observed, to ensure good health, and acclimatized for at least 5 days prior to the experiment and treated in accordance with the European Convention for the Protection of Vertebrate Animals used for Experimental and Other Scientific Purposes. The animals were fed twice daily with standard commercial pig feed. Food was withdrawn 12 h prior to the experiment. Water was provided ad libitum. All animals were housed under standardized lightning (16:8 h light:dark cycle), temperature (20°C), and humidity (25% RH).

### Anesthesia

The premedication was chosen based on cardiovascular stability and the pigs were premedicated with 10 mg diazepam (Stesolid®, Actavis Nordic AS, Gentofte Denmark), 500 mg ketamine (Ketalar®, Pfizer AB, Sollentuna Sweden), and 1 mg atropine (Atropin®, Nycomed Pharma AS, Asker Norway) as an intramuscular injection 15–30 min prior to weighting and induction of anesthesia.

After cannulation of an auricular vein, anesthesia was induced with a supplement intravenous injection of 125 mg thiopentone (Pentocur®, Abcur AB, Helsingborg Sweden), 250 mg ketamine (Ketalar®, Pfizer AB, Sollentuna Sweden), and 250 *μ*g fentanyl (Fentanyl‐Hameln®, Hameln Pharmaceuticals GmbH, Hameln Germany). After one tracheal spray with 5 ml 4% lidocaine (Ullevaal hospital pharmacy, Oslo), the pigs were intubated in right lateral position. The mechanical ventilator (Aisys®, Datex‐Ohmeda, GE Healthcare) was set to volume‐controlled modus; tidal volume 10 mL/kg, respiration rate was adjusted to maintain PaCO_2_ at 4.5–5.5 kPa (34–42 mmHg) and the fraction of inspired oxygen (FiO_2_) was kept at 0.4. Anesthesia was maintained by a continuous infusion of 0.42 mg kg^−1^ h^−1^ midazolam (Midazolam®, Rotexmedica GmbH, Trittau Germany), 20 *μ*g kg^−1^ h^−1^ fentanyl (Fentanyl‐Hameln®, Hameln Pharmaceuticals GmbH, Hameln Germany), and 4 mg kg^−1^ h^−1^ Thiopentone (Pentocur®, Abcur AB, Helsingborg Sweden). If the animal showed signs of shivering, we gave an additional 250 *μ*g fentanyl. During the surgical phase of this experiment, the anesthesia was supplemented with 0.5–1.0% isoflurane (Isofluran®, Baxter, Norway). Fluid balance was achieved using a continuous infusion of normal saline (NaCl 9 mg/mL, Fresenius Kabi, Upsala Sweden) at 10 mL kg^−1^ h^−1^. The temperature was kept 38–39°C using a heating pad. Heart rate was measured using surface ECG electrodes and level of saturated oxygen (SpO_2_) measured by a tail sensor. Cardiac output was measured with microspheres using the reference blood sample technique (Rudolph and Heymann 1967).

### Invasive procedures

To preserve a closed chest, minimal traumatic model, all catheters and cannulation of arteries and veins were made through a mini‐invasive surgical cut‐down, where the incisions were made as small as possible. Before invasive procedures took place the pig was given intravenous 1 g cephalotin (Cephalotin®, Willerton Invest SA, Luxembourg) as a prophylactic. A pulmonary artery catheter (Swan‐Ganz CCOmbo®, Edward Lifescience) was introduced at the right internal jugular vein and the tip of the catheter was positioned in the pulmonary artery guided by typical pressure waves. The carotid artery was reached from a left neck incision and a kinked Portex catheter (4 Fr., 30 cm, Portex Intravenous Cannula®, Sims Portex Ltd, UK) for injection microspheres was inserted into the artery and advanced retrograde into the apex of the left ventricle guided by typical pressure waves. A three‐lumen central venous cannula (Certofix Trio, Braun) was inserted in the deep right femoral artery and advanced up to the descending aorta for invasive blood pressure measurements, blood gas sampling, reference blood sample during microsphere injection, and exsanguination.

Six microdialysis catheters (CMA 20 Elite Microdialysis Probe®, CMA Microdialysis, Solna Sweden) were inserted through a small surgical incision, into the tissue, or on the surface of the three studied organs; two probes were placed between the capsule and the surface of the right kidney, two probes were placed into the gut lumen with close contact with the mucosa of the ileum, and two into the belly of the gracilis muscle. A urinary bladder catheter was inserted via a midline minilaparotomy and urine output measured every hour. The pig was during the surgical phase laid in a supine position.

All animals were thereafter allowed at least 60 min stabilization before the experiment started. During this period, isoflurane was discontinued and washed out.

In several animals, a biosensor from another research group were placed in the left superficial femoral artery and tested during the experiment (N. A. Elvemo, unpublished data). This was done without interference with this study.

### Regional blood flow measurement

Fluorescent‐labeled polystyrene microspheres, 15 *μ*m diameter, in four colors (Dye‐Trak® “F” fluorescent microspheres, Triton Technologies Inc., CA) were stored shielded from long‐term exposure to light to prevent loss of fluorescence intensity. For each blood flow measurement, 6.0 × 10^6^ microspheres suspended in 6 mL of 0.9% saline containing 0.02% Tween 80 were aspirated into a syringe and injected over 30 sec into the left ventricle. Before aspirating and injecting, the stock solution was thoroughly sonicated (5 min) and vortexed (3 min). The injection of microspheres was followed by two 6 mL normal saline flushes over a period of 30 sec. Before each injection, the correct position of the ventricular catheter was verified by typical pressure waves. An arterial reference blood sample from the descending aorta was withdrawn by a retracting pump (Cole‐Palmer Instrument Company, 74900 series) at a rate of 4 mL/min, starting 15 sec before and continued for 120 sec after the microsphere injection. The order in which the different colored microspheres were injected was randomized.

After euthanasia, we harvested two samples, which lay in vicinity of the microdialysis probe placement, from each organ of interest. All the connective tissue was carefully removed, and kidney samples were only from cortex, while samples from ileum contained only mucosa. Both organ samples and reference blood samples were weighted and dissolved over night in 4 mol/L KOH. Further recovery of microspheres from tissue and blood samples was done with negative pressure filtration using 10 *μ*m membrane filters, (Omnipore®, Merck Millipore, Ireland) according to the manufacturer's extraction protocol (Triton Technologies Inc., California). Fluorescence intensity, which indirectly represents number of microspheres in the sample, was measured with a microplate reader (Fluostar Omega, BMG Labtech GmbH, Germany).

Regional blood flow to each of obtained organs was determined by the reference blood sample technique (Glenny et al. 1985).

### Regional metabolic stress response measurement

We used the CMA 20 Elite Probe^®^ (CMA Microdialysis, Solna Sweden) which has a 10‐mm long probe membrane with a 20 kD molecular cut‐off. All the catheters were perfused at a flow rate of 1 *μ*L/min with Perfusion fluid T1® (M Dialysis AB, Stockholm, Sweden). After placement, all catheters were perfused in situ for at least 60 min during the stabilization period to establish reliable equilibration with the surrounding tissue. Samples from each microdialysis catheter were collected over 20 min in microvials and analyzed on‐site for lactate, pyruvate, glucose, and glycerol concentrations (Iscus® Microdialysis Analyzer, CMA Microdialysis AB, Solna, Sweden). Each sampling started 10 min before and continued 10 min after fluorescence‐labeled microsphere injection. Care was taken to try to place the catheter close to the location where the predetermined biopsies would be taken.

### Experimental protocol

Baseline values were obtained after the stabilization period. Every 5 min during the experiment heart rate, blood pressure, pulmonary artery pressure, and peripheral oxygen saturation were measured. After baseline values had been obtained, hypovolemia by exsanguination was induced by withdrawal of blood (35 mL/kg body weight, which correspond to 50% of estimated total blood volume of 70 mL/kg) over 30 min from descending aorta. The blood was collected in a heparinized bag (7 mL Heparin 5000 IU/mL) and kept warm in an Incubator shaker (37°C, 50 rpm) for autologous transfusion 70 min after completed exsanguination.

Regional blood flow and regional metabolism were sampled four times during the experiment; baseline values and then after 50, 100, and 150 min. The microdialysate were collected over a period of 20 min and microspheres were injected 10 min into this process. Arterial and mixed venous blood gas were obtained and analyzed (ABL 725® Bergman Diagnostika, Radiometer, Copenhagen, Denmark) seven times; baseline and after 50, 85,100, 150, 180, and 200 min. Urine output was measured every hour.

After 220 min, the pig was euthanized with a lethal dose of saturated potassium chloride (20 mL KCl). The following organs were harvested; the right kidney, the ileum and the gracilis muscle. During dissection, proper placement of the microdialysis probe were confirmed.

### Statistical analysis

Statistical analyses were made with the statistical software “R” with the *nlme* package for mixed effect modeling (R Development Core Team, 2011; Pinheiro et al. 2015). According to the nature of the analyses, either a categorical or a continuous variable were used as fixed effects, and the influence of individual animals as random effect. In addition, we added an autocorrelation coefficient to account for the serial nature of the measurements (AR1‐process). We compared the models with the null model by a likelihood ratio test (all models with *P* < 0.05). Data from regional blood flow and regional metabolism were sampled four times during the experiment, and therefore analyzed as categorical, belonging to one of the four levels; baseline (BL) at 0 min, early hemorrhagic (EH) at 50 min, late hemorrhagic (LH) at 110 min, or retransfused (RT) at 160 min. Individual *P*‐values for the different levels of the categorical predictor were extracted from the macrocirculatory and microdialysis data by the *gmodels* package (Warnes et al. 2015). Values of *P* < 0.05 are reported in the text. If not otherwise stated, all results are reported as means ± standard deviation (SD).

## Results

Of nine pigs, where one was excluded due to technical problems and one died short after exsanguination, seven were included in the analysis and results.

### Macrocirculatory changes

**Figure 1:**
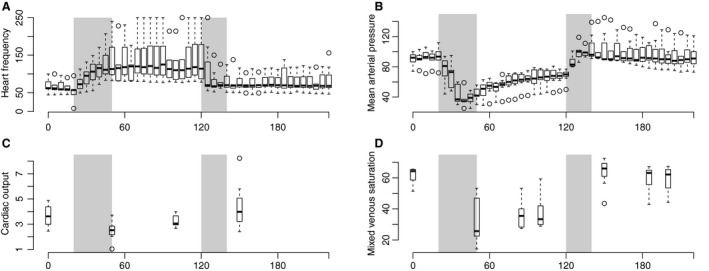
Boxplot of macrocirculatory changes. Periods of bleeding and retransfusion marked with gray area. (A) Heart rate (B) Mean arterial pressure (C) Cardiac output (D) Mixed venous saturation. Note different scales on individual axes.

The baseline heart rate (HR) was 66.2 (±15.6) beats per minute and the mean arterial pressure (MAP) 89.3 (±5.3) mmHg. During the early hemorrhagic period, HR rose steeply to 141.1 (±11.0) bpm and remained at this level until transfusion whereupon it fell back to its baseline level, whereas MAP dropped to 38.0 (±4.6) mmHg during early hemorrhagic, but started to rise gradually on its own during the late hemorrhagic phase. During transfusion MAP increased steeply to 105.0 (±5.0) mmHg before it gradually decreased toward baseline level at the end of the experiment (Fig. 1A and B).

Cardiac output (CO) and mixed venous saturation (SvO_2_) showed a similar trend with an abrupt fall from baseline 3.7 (±0.5) L/min and 61.3 (± 3.9)% to 2.5 (±0.6) L/min and 33.2 (±3.3)% during the early hemorrhagic phase, with a gradual increase during the late hemorrhagic phase until it rose steeply after transfusion to 4.5 (±0.6) L/min and 63.2 (±4.3)% (Fig. 1 C and D).

**Figure 2:**
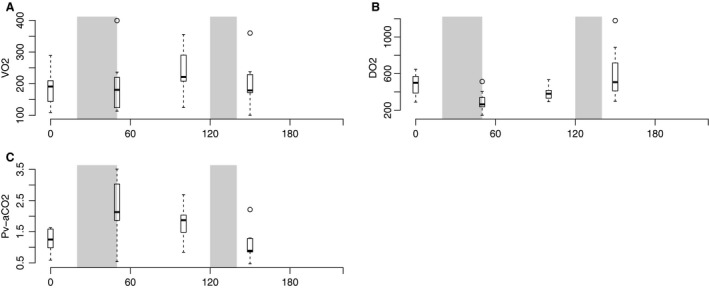
Boxplot of changes in global oxygen balance and venous‐to‐arterial carbon dioxide difference. Periods of bleeding and retransfusion marked with gray area. (A) Global oxygen consumption (*V*O_2_), (B) Global oxygen deliverance (DO_2_), (C) Systemic arterial‐to‐venous carbon dioxide difference (Pv‐aCO_2_). Refer to text for individual changes with *P* < 0.05. Note different scales on individual axes.

Oxygen consumption (*V*O_2_) was stable during the whole experiment with a slight increase from its baseline value 185.2 (±30.5) mL/min to a maximum of 242.5 (±27.2) mL/min, whereas oxygen deliverance (DO_2_) showed a similar curve to CO with an abrupt fall from baseline 478.5 (±74.6) mL/min to 296.7 (±71.1) mL/min after exsanguination and a gradual increase during the late hemorrhagic phase until a steep rise after transfusion to 605.6 (±90.1) mL/min (Fig. 2 A and B).

Pv‐aCO_2_ rose briskly from its baseline value 1.23 (±0.40) kPa to a maximum 2.28 (±1.02) kPa in the early hemorrhagic period, but started to decline to 1.77 (±0.58) kPa in the late hemorrhagic period. This trend accelerated after transfusion to 1.12 (±0.57) kPa (Fig. 2C, all changes with a *P* < 0.05. Baseline was not significantly different from after transfusion).

Hemoglobin concentration (Hgb) fell slightly from its baseline value 9.7 (±0.40) g/dl to a nadir of 8.9 (±0.25) g/dl in the late hemorrhagic phase and thereafter returned to its baseline value after transfusion (data not shown).

Systemic acid–base measurements did not deviate much from the baseline pH value of 7.46 (± 0.02) during the experiment. Systemic lactate increased from baseline 0.70 (± 0.17) mmol/l to 1.97 (± 0.16) mmol/l during early hemorrhagic and thereafter returned to baseline starting in the late hemorrhagic phase. Base excess (BE) showed a similar pattern with a decrease from baseline + 5.21 (± 0.83) to a nadir of + 1.7 (± 0.39) before returning to baseline (data not shown).

### Microcirculatory and tissue metabolic changes

**Figure 3:**
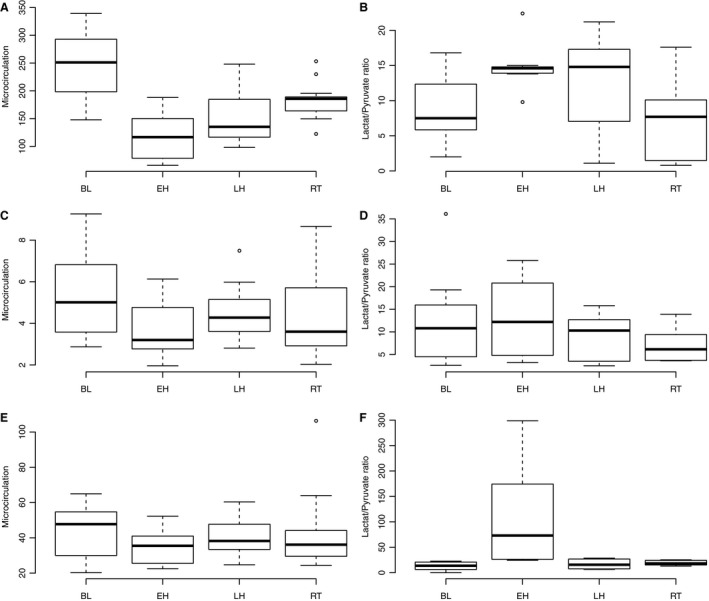
Boxplot of microcirculatory and metabolic tissue changes. (A) Kidney microcirculation (B) Kidney lactate/pyruvate ratio (C) Muscle microcirculation (D) Muscle lactate/pyruvate ratio (E) Gut microcirculation (F) Gut lactate/pyruvate ratio. Refer to text for individual changes with *P* < 0.05. Note different scales on individual axes. (BL baseline, 0 min; EH, early hemorrhage, 50 min; LH, late hemorrhage, 100 min; RT, re‐transfused, 150 min). BL, baseline; EH, early hemorrhagic; RT, retransfused.

In the cortex of the kidneys, blood flow decreased abruptly from baseline 246.5 (±13.9) mL min^−1^ 100 g^−1^ to 114.2 (±17.0) during early hemorrhagic phase, but started gradually to increase during the late hemorrhagic phase to 154.0 mL min^−1^ 100 g^−1^ (±16.8) and after transfusion to a value near its baseline value of 180.0 (±17.2) mL min^−1^ 100 g^−1^ (Fig. 3A, all changes with *P* < 0.05). The lactate/pyruvate (L/P) ratio reflected the induced ischemia with high L/P‐values at periods with low flow, with the respective values of 9.0 (±2.2), 14.9 (±2.5), 12.3 (±3.0), and 7.0 (±3.1) (Fig. 3B, change from “BL” to “EH” and “EH” to “RT” with *P* < 0.05).

In the gracilis muscle, the mean blood flow decreased slightly after exsanguination from 5.12 mL min^−1^ 100 g^−1^ (±0.53) to 3.66 mL min^−1^ 100 g^−1^ (±0.45) and rose gradually during the late hemorrhagic phase to 4.46 mL min^−1^ 100 g^−1^ (±0.47) and after transfusion to 4.40 mL min^−1^ 100 g^−1^ (±0.47) (Fig. 3C, change from “BL” to “LH” with *P* < 0.05). The mean L/P‐ratio rose after exsanguination from 12.9 (±4.0) to 16.6 (±2.6) and started to decrease toward baseline level during the late hemorrhagic phase to 12.2 (±2.8), the decrease continued gradually after transfusion to 9.8 (±3.3) (Fig. 3D, change from “EH” to “LH” and “EH” to “RT” with *P* < 0.05).

In the mucosa of the ileum, mean blood flow decreased slightly after exsanguination from 43.4 mL min^−1^ 100 g^−1^ (±4.7) to 34.7 mL min^−1^ 100 g^−1^ (±4.0) and increased slightly during the late hemorrhagic phase to 40.7 mL min^−1^ 100 g^−1^ (±4.5) and after transfusion to 42.4 mL min^−1^ 100 g^−1^ (±4.4) (Fig. 3E, change from “BL” to “EH” and “EH” to “RT” with *P* < 0.05). Mean L/P‐ratio increased steeply after exsanguination from 12.9 (±23.8) to 111.7 (± 32.4) but was almost restored during the late hemorrhagic phase to 16.1 (±33.7), after transfusion the mean L/P‐ratio decreased slightly to 17.8 (±33.7) (Fig. 3F, change from “BL” to “EH”, “EH” to “LH”, and “EH” to “RT” with *P* < 0.05).

## Discussion

This study aimed at describing the effects of severe blood loss on the macrocirculation, the microcirculation, and regional metabolism in the absence of tissue trauma. We found an overall coherence in the changes between the measured variables.

The severe exsanguination in this experiment led to anticipated macrocirculatory effects, that is, reduced CO and MAP with a compensatory rise in HR. Thus, global oxygen deliverance, DO_2_, was seriously compromised, and with an unaltered consumption, *V*O_2_, SvO_2_ dropped to a low level. The fall in CO leads to a fall in microcirculatory blood flow and a rise in tissue L/P‐ratio. In the late hemorrhagic period, before blood was re‐transfused, both the macro‐ and microcirculatory variables started to improve spontaneously and gradually. This might be due to adrenergic venoconstriction of the unstressed venous blood, pooled in capacitance vessels in the abdominal organs (Greenway and Lautt 1986; Ross et al. 2014). Some hemorrhagic models involves splenectomizing of the animals prior to exsanguination to blunt this response (Larentzakis et al. 2013), but the benefit of this is debatable and might introduce a bias (Boysen et al. 2016). However, the SvO_2_ remained low, maybe due to an oxygen debt from the early exsanguination period.

We used increase in L/P‐ratio and systemic lactate levels as markers of a stressed metabolism. Although all animal cells will produce lactate during anaerobic conditions, the etiology of lactataemia is complex. It has been shown that increased levels of catecholamine induces glycolysis to a level where pyruvate production exceeds its utilization by the mitochondria so that lactate is produced regardless of aerobic conditions (Levy 2006; Marik and Bellomo 2013). It has also been shown that this adrenergic induced hypermetabolism is more common in shock states (McCarter et al. 2001; Levy et al. 2008). Regardless of its origin, increased lactate level, and especially lack of lactate clearance, is well established markers of severity and mortality in critically ill patients (Nguyen et al. 2004; Mikkelsen et al. 2009; Baxter et al. 2016).

The venous‐to‐arterial carbon dioxide difference (Pv‐aCO_2_) increases earlier than lactate with a falling CO. A sustained increased Pv‐aCO_2_ difference in spite of a sufficient CO is interpreted as a sign of microcirculatory derangement (Vallet et al. 1985; Ospina‐Tascón et al. 2016b). In a recent study of septic shock, alterations in Pv‐aCO_2_ differences reflected adequacy of the microcirculation evaluated with a sidestream dark‐field device (Ospina‐Tascón et al. 2016a). In our model, Pv‐aCO_2_ increased with a falling CO and SvO_2_ after exsanguination, and fell to its baseline value after the transfusion normalized both CO and SvO_2_. This finding also supports our main conclusion of an overall consistency of coherence between changes in macrocirculatory indices, microcirculatory blood flow and tissue metabolic response.

After retransfusion, HR normalized, and MAP, CO, SvO_2_, and DO_2_ rose to a higher level than baseline. In the kidney cortex, retransfusion increased regional blood flow, albeit not to baseline values, while the L/P‐ratio continued to drop to a lower level than baseline. The overshoot and undershoot from baseline in, respectively, macro‐ and microcirculatory variables could be evidence of an adrenergic vasoconstriction that increased preload and afterload, but limited regional organ flow. At the same time, the L/P‐ratio in the kidney decreased to a lower level than baseline, which could be due to less stressed metabolism. There has been a discussion on the role of exogenous vasopressors in the resuscitation of patients in traumatic hemorrhagic shock, due to the development of an inflammatory response and that endogen sympathetic support is exhausted and tapers over time (Beloncle et al. 2013; Harrois et al. 2014). The result is often described as an end‐stage of hemorrhagic shock with concomitant bradycardia and cardiovascular collapse. In this model, both spontaneously improvement during hypovolemic phase in macrocirculatory variables and then an overshoot after transfusion suggest that the pigs still had intact sympathetic reserves.

Our study did not confirm findings from a previous study using sidestream dark‐field imaging device during traumatic hemorrhagic shock where the microcirculation was impaired despite correction of macrocirculatory values (Tachon et al. 2014), but our study confirms the results from other similar study of minimal‐traumatic hemorrhagic shock where the microcirculation followed the macrocirculation both during exsanguination and transfusion (Van Iterson et al. 2012). The most common cause for severe hemorrhage is trauma, which induces an inflammatory response on its own (Gierer et al. 2008), and, as mentioned in the introduction, we speculate that the lack of trauma in this model might be the reason for the lack of macro‐ and microcirculatory discrepancy and the lack of sustained local metabolic stress response. Age and comorbidities are also factors that make an individual more prone to the deleterious effects of shock (Morris et al. 1990; Taylor et al. 2002). In our study, we used healthy juvenile pigs that showed a remarkable ability to restore homeostasis after exsanguination. Finally, we presume that the deep level of anesthesia, which induces dilatation of constricted vascular beds and lowered organ oxygen demand (*V*O_2_), might have protected the animals. The rise in systemic lactate and fall in SvO_2_ levels (as respective markers of systemic metabolic stress response and oxygen consumption) in our model are only modest compared to a comparable exsanguination in conscious pigs of equal size (Hannon et al. 1990). At the same time, we believe that withdrawal of 50% of estimated total blood volume (70 mL/kg) is sufficient to produce a severe hemorrhagic shock. This claim is supported by that one of nine pigs became extremely circulatory unstable and died short after exsanguination. Furthermore, in a comparable study to detect organ ischemia in pigs during hemorrhagic shock, Kvarstein et al. (2003) found that median critical blood loss was ~40% of estimated total blood volume (70 mL/kg).

As always with large animal studies, the size, prize, and labor with the models yield the problem of under‐powering the studies. However, large animal studies should always aim at showing some general qualitative physiological feature rather than a quantification of the changes, and as such we believe we have succeeded and that our study size is sufficient. Furthermore, results from animal studies might not translate to humans and should therefore always be interpreted with care. The use of fluorescent‐labeled polystyrene microspheres and microdialysis are both complicated techniques and can induce measurement error on their own. To what extent these methods represents changes on a cellular level is uncertain; the microsphere technique cannot detect shunting of the blood flow within the obtained sample, as long as total blood flow is preserved, and the microdialysis catheter only capture samples only in close vicinity of the membrane. Although, these methods allow researchers to evaluate circulation and metabolism on a smaller scale, it is still a measurement of overall relative changes in a region of an organ. There has been concern if repeated injections of polystyrene microspheres could successively clot the capillaries and bias subsequent measures of regional flow. However, extensive research has shown that, within dose limits and injected systemically, the microsphere technique does not significantly obstruct capillary flow (Prinzen and Bassingthwaighte 2000; Bartoli et al. 2008). We chose to place the microdialysis catheters, which monitored the kidney and the gut, on the surface and the lumen of the organs. These placements have been shown to mirror the L/P‐ratio changes within liver, heart and gut mucosa without injuring the organs (Solligård et al. 2004; Abrahamsson et al. 2011, 2012). However, not placing the catheter in the parenchyma may blunt and/or delay changes in the L/P‐ratio and therefore introduce an error.

## Conclusion

In conclusion, we found in this study of hemorrhagic shock and retransfusion in pigs an overall consistency of coherence between changes in macrocirculatory indices, microcirculatory blood flow and tissue metabolic response. This indicates that severe, but brief, hemorrhage with minimal tissue injury is in itself not sufficient to cause lack of coherence between macro‐ and microcirculation.

## Conflict of Interests

The authors declare that they have no competing interests.

## References

[phy213216-bib-0001] Abrahamsson, P. , A.‐M. Aberg , G. Johansson , O. Winsö , A. Waldenström , and M. Haney . 2011 Detection of myocardial ischaemia using surface microdialysis on the beating heart. Clin. Physiol. Funct. Imaging 31:175–181. 21091606 10.1111/j.1475-097X.2010.00995.x

[phy213216-bib-0002] Abrahamsson, P. , A.‐M. Aberg , O. Winsö , G. Johansson , M. Haney , and P.‐J. Blind . 2012 Surface microdialysis sampling: a new approach described in a liver ischaemia model. Clin. Physiol. Funct. Imaging 32:99–105. 22296629 10.1111/j.1475-097X.2011.01061.x

[phy213216-bib-0003] Bartoli, C. R. , K. Okabe , I. Akiyama , B. Coull , and J. J. Godleski . 2008 Repeat microsphere delivery for serial measurement of regional blood perfusion in the chronically instrumented, conscious canine. J. Surg. Res. 145:135–141. 17632127 10.1016/j.jss.2007.04.012 PMC2277484

[phy213216-bib-0004] Baxter, J. , K. R. Cranfield , G. E. Clark , T. Harris , B. Bloom , and A. J. Gray . 2016 Do lactate levels in the emergency department predict outcome in adult trauma patients? a systematic review. J Trauma Acute Care Surg. 81:555–566. 27280943 10.1097/TA.0000000000001156

[phy213216-bib-0005] Beloncle, F. , F. Meziani , N. Lerolle , P. Radermacher , and P. Asfar . 2013 Does vasopressor therapy have an indication in hemorrhagic shock? Ann. Intensive Care 3:13. 23697682 10.1186/2110-5820-3-13 PMC3691630

[phy213216-bib-0006] Boysen, S. R. , N. A. Caulkett , C. E. Brookfield , A. Warren , and J. M. Pang . 2016 Splenectomy versus sham splenectomy in a swine model of controlled hemorrhagic shock. Shock 46:439–446. 26974424 10.1097/SHK.0000000000000608

[phy213216-bib-0007] De Backer, D. , D Orbegozo Cortes K. Donadello , and J‐L Vincent . 2014 Pathophysiology of microcirculatory dysfunction and the pathogenesis of septic shock. Virulence 5:73–79. 24067428 10.4161/viru.26482 PMC3916386

[phy213216-bib-0008] Gierer, P. , J. N. Hoffmann , F. Mahr , M. D. Menger , T. Mittlmeier , G. Gradl , et al. 2008 Sublethal trauma model with systemic endotoxemia for the study of microcirculatory disorders after the second hit. J. Surg. Res. 147:68–74. 18028956 10.1016/j.jss.2007.07.025

[phy213216-bib-0009] Glenny, R. W. , S. Bernard , and M. Brinkley . 1985 Validation of fluorescent‐labeled microspheres for measurement of regional organ perfusion. J. Appl. Physiol. (Bethesda Md) 1993:2585–2597. 10.1152/jappl.1993.74.5.2585 8335595

[phy213216-bib-0010] Greenway, C. V. , and W. W. Lautt . 1986 Blood volume, the venous system, preload, and cardiac output. Can. J. Physiol. Pharmacol. 64:383–387. 3730923 10.1139/y86-062

[phy213216-bib-0011] Hannon, J. P. , C. E. Wade , C. A. Bossone , M. M. Hunt , R. I. Coppes , and J. A. Loveday . 1990 Blood gas and acid‐base status of conscious pigs subjected to fixed‐volume hemorrhage and resuscitated with hypertonic saline dextran. Circ. Shock. 32:19–29. 1698570

[phy213216-bib-0012] Harrois, A. , S. R. Hamada , and J. Duranteau . 2014 Fluid resuscitation and vasopressors in severe trauma patients. Curr. Opin. Crit. Care 20:632–637. 25340381 10.1097/MCC.0000000000000159

[phy213216-bib-0013] Ince, C. 2004 Microcirculation in distress: a new resuscitation end point? Crit. Care Med. 32:1963–1964. 15343029 10.1097/01.ccm.0000139617.88704.b9

[phy213216-bib-0014] Kvarstein, G. , P. Mirtaheri , and T. I. Tønnessen . 2003 Detection of organ ischemia during hemorrhagic shock. Acta Anaesthesiol. Scand. 47:675–686. 12803584 10.1034/j.1399-6576.2003.00134.x

[phy213216-bib-0015] Larentzakis, A. , K. G. Toutouzas , A. Papalois , G. Lapidakis , S. Doulgerakis , G. Doulami , et al. 2013 Porcine model of hemorrhagic shock with microdialysis monitoring. J. Surg. Res. 179:e177–e182. 22480841 10.1016/j.jss.2012.01.040

[phy213216-bib-0016] Levy, B. 2006 Lactate and shock state: the metabolic view. Curr. Opin. Crit. Care 12:315–321. 16810041 10.1097/01.ccx.0000235208.77450.15

[phy213216-bib-0017] Levy, B. , O. Desebbe , C. Montemont , and S. Gibot . 2008 Increased aerobic glycolysis through beta2 stimulation is a common mechanism involved in lactate formation during shock states. Shock 30:417–421. 18323749 10.1097/SHK.0b013e318167378f

[phy213216-bib-0018] Lord, J. M. , M. J. Midwinter , Y.‐F. Chen , A. Belli , K. Brohi , E. J. Kovacs , et al. 2014 The systemic immune response to trauma: an overview of pathophysiology and treatment. Lancet 384:1455–1465. 25390327 10.1016/S0140-6736(14)60687-5 PMC4729362

[phy213216-bib-0019] Marik, P. E. , R. Bellomo . 2013 Lactate clearance as a target of therapy in sepsis: a flawed paradigm. OA Crit. Care 1:3.

[phy213216-bib-0020] McCarter, F. D. , J. H. James , F. A. Luchette , L. Wang , L. A. Friend , J. K. King , et al. 2001 Adrenergic blockade reduces skeletal muscle glycolysis and Na(+), K(+)‐ATPase activity during hemorrhage. J. Surg. Res. 99:235–244. 11469892 10.1006/jsre.2001.6175

[phy213216-bib-0021] Mikkelsen, M. E. , A. N. Miltiades , D. F. Gaieski , M. Goyal , B. D. Fuchs , C. V. Shah , et al. 2009 Serum lactate is associated with mortality in severe sepsis independent of organ failure and shock. Crit. Care Med. 37:1670–1677. 19325467 10.1097/CCM.0b013e31819fcf68

[phy213216-bib-0022] Morris, J. A. Jr. , E. J. MacKenzie , and S. L. Edelstein . 1990 The effect of preexisting conditions on mortality in trauma patients. JAMA 263:1942–1946. 2313871

[phy213216-bib-0023] Nguyen, H. B. , E. P. Rivers , B. P. Knoblich , G. Jacobsen , A. Muzzin , J. A. Ressler , et al. 2004 Early lactate clearance is associated with improved outcome in severe sepsis and septic shock. Crit. Care Med. 32:1637–1642. 15286537 10.1097/01.ccm.0000132904.35713.a7

[phy213216-bib-0024] Ospina‐Tascón, G. A. , M. Umaña , W. F. Bermúdez , D. F. Bautista‐Rincón , J. D. Valencia , H. J. Madriñán , et al. 2016a Can venous‐to‐arterial carbon dioxide differences reflect microcirculatory alterations in patients with septic shock? Intensive Care Med. 42:211–221. 26578172 10.1007/s00134-015-4133-2 PMC4726723

[phy213216-bib-0025] Ospina‐Tascón, G. A. , G. Hernández , and M. Cecconi . 2016b Understanding the venous‐arterial CO_2_ to arterial‐venous O_2_ content difference ratio. Intensive Care Med. 42:1801–1804. 26873834 10.1007/s00134-016-4233-7

[phy213216-bib-0026] Pinheiro, J. , D. Bates , S. DebRoy , and D. Sakar ; R Core Team . 2015. nlme: Linear and Nonlinear Mixed Effects Models.

[phy213216-bib-0027] Prinzen, F. W. , and J. B. Bassingthwaighte . 2000 Blood flow distributions by microsphere deposition methods. Cardiovasc. Res. 45:13–21. 10728307 10.1016/s0008-6363(99)00252-7 PMC3483311

[phy213216-bib-0028] R Development Core Team . 2011 R: A Language and Environment for Statistical Computing: the R Foundation for Statistical Computing. [Internet]. 2011th ed. Vienna, Austria; Available at: http://www.R-project.org/.(accessed 14 September 2016).

[phy213216-bib-0029] Ross, J. D. , C. J. Burns , E. M. Sagini , L.‐A. Zarzabal , and J. J. Morrison . 2014 A laparoscopic swine model of noncompressible torso hemorrhage. J. Trauma Acute Care Surg. 77:S77–S82. 25159366 10.1097/TA.0000000000000385

[phy213216-bib-0030] Rudolph, A. M. , and M. A. Heymann . 1967 The circulation of the fetus in utero. Methods for studying distribution of blood flow, cardiac output and organ blood flow. Circ. Res. 21:163–184. 4952708 10.1161/01.res.21.2.163

[phy213216-bib-0031] Solligård, E. , I. S. Juel , K. Bakkelund , H. Johnsen , O. D. Saether , J. E. Grønbech , et al. 2004 Gut barrier dysfunction as detected by intestinal luminal microdialysis. Intensive Care Med. 30:1188–1194. 14991095 10.1007/s00134-004-2173-0

[phy213216-bib-0032] Spronk, P. E. , D. F. Zandstra , and C. Ince . 2004 Bench‐to‐bedside review: sepsis is a disease of the microcirculation. Crit. Care Lond. Engl. 8:462–468. 10.1186/cc2894 PMC1065042 15566617

[phy213216-bib-0033] Tachon, G. , A. Harrois , S. Tanaka , H. Kato , O. Huet , J. Pottecher , et al. 2014 Microcirculatory alterations in traumatic hemorrhagic shock. Crit. Care Med. 42:1433–1441. 24561562 10.1097/CCM.0000000000000223

[phy213216-bib-0034] Taylor, M. D. , J. K. Tracy , W. Meyer , M. Pasquale , and L. M. Napolitano . 2002 Trauma in the elderly: intensive care unit resource use and outcome. J. Trauma 53:407–414. 12352472 10.1097/00005373-200209000-00001

[phy213216-bib-0035] Vallet, B. , J. L. Teboul , S. Cain , and S. Curtis . 1985 Venoarterial CO(2) difference during regional ischemic or hypoxic hypoxia. J. Appl. Physiol.(Bethesda Md)2000:1317–1321. 10.1152/jappl.2000.89.4.1317 11007564

[phy213216-bib-0036] Van Iterson, M. , R. Bezemer , M. Heger , M. Siegemund , and C. Ince . 2012 Microcirculation follows macrocirculation in heart and gut in the acute phase of hemorrhagic shock and isovolemic autologous whole blood resuscitation in pigs. Transfusion (Paris). 52:1552–1559. 10.1111/j.1537-2995.2011.03471.x 22168283

[phy213216-bib-0037] Vincent, J.‐L. , and D. De Backer . 2014 Circulatory shock. N. Engl. J. Med. 370:583. 10.1056/NEJMc1314999 24499231

[phy213216-bib-0038] Warnes, G. R. , B. Bolker , T. Lumley . 2015 SAIC‐Frederick and RCJC from RCJ are C (2005), ResearchProgram IF by the I, NIH of the, et al. gmodels: Various R Programming Tools for Model Fitting [Internet]. Available at: https://cran.r-project.org/web/packages/gmodels/index.html (accessed 14 September 2016)

